# The Value of X-Ray Therapy for Metastases in Bone from Carcinoma of the Breast
*An address to the Bristol Medico-Chirurgical Society, on Wednesday, April 12th, 1950.


**Published:** 1950-07

**Authors:** Sydney Curwen

**Affiliations:** Radiotherapist, United Bristol Hospitals


### THE VALUE OF X-RAY THERAPY FOR METASTASES IN BONE FROM CARCINOMA OF THE BREAST*

BY

SYDNEY CURWEN, D.M.R.T.

*Radiotherapist, United Bristol Hospitals.*

It is my object in presenting this short paper to emphasize the value of this method for the treatment of bone metastases, which occur all too frequently as a complication of carcinoma of the breast. A problem of this sort should not be approached in a spirit of competition. Cancer is not the field of the radiotherapist alone any more than it is that of the surgeon, or of any other clinician alone. A patient suffering from cancer should be the subject of careful consultation by all those who can help to palliate or cure the disease.

When a woman, who has had the breast removed for carcinoma, experiences pain in or referred to bone, it is usually the first sounding of the death knell. The *average* survival even in treated cases is little more than a year. The surgeon can do nothing other than try various measures for the destruction of nerves. The physician is powerless except for the use of endocrines, and these preparations are in their infancy. It should be mentioned, however, in passing, that the results of hormone therapy have been remarkable in some cases, and should spur research workers to greater efforts.

At the moment the main responsibility in cases of carcinoma of the breast falls on the general practitioner. Osler in 1906 wrote as follows: " Scarcely enough stress is laid in the works of surgery upon the terminal stages of cancer with the tragic events of which the general practitioner is left to battle alone. . . . The most common and the most serious, as entailing a maximum, are the lesions of the spine. Amid the sad failures of our art these cases stand out in strong relief. An early diagnosis of the condition (bone metastasis) and an early

* An address to the Bristol Medico-Chirurgical Society, on Wednesday, April 12th, 1950.

recognition of the hopelessness may lessen the terrible ordeal of the poor victim, and mitigate not only her suffering, but that of her relatives. Morphine, enough, given freely and without fear, in doses sufficient to keep the patient comfortable, affords the only possible relief."

That view has been held for many years and has adherents even to-day. It is my firm belief, however, that in cases of bone metastases the best relief from suffering can be derived from X-ray therapy. Ewing states in his *Neoplastic Diseases*, "As a palliative method radiation causes complete regression of many local recurrences. Some of the most striking results are seen in the control of bone metastases in which pain is relieved, bone structure restored and life prolonged".

The symptomatology is typical, but it should be remembered that secondaries demonstrable by X-rays may be present for many months, or even years, before giving rise to symptoms. It is economically impossible for X-ray pictures to be taken of every bone each time patients attend for follow-up. In any case a procedure of that sort would not be necessarily desirable from the patients' point of view.

The most frequent first symptom to bring the patient for advice, or which she may mention during her routine follow-up, is pain. Two types of pain are described: (1) Local, originating through pressure on the periosteum; or (2) due to pressure on sensory nerves and involving their distribution.

The symptoms may be described by the patient as neuralgia or rheumatism. At the outset the pain may be mild and transitory, being described as a stab or stitch. There may be hypersensitivity to jarring movements. As the pain becomes more severe it may be agonizing or crippling. Sometimes a pathological fracture is the first indication. In the early stages the patient's general condition is fairly good.

*Diagnosis*. Symptoms are very suggestive, and in every case where pain is described X-ray pictures should be taken. These show various changes.

1. Seventy-five per cent. are osteolytic in type; the picture is one of bone erosion and loss of normal trabeculation, giving rise to a sharply defined or diffuse area of rarefaction.

2. Osteoblastic, bone density increased.

3. A mixed type.

4. No X-ray evidence at first, but the typical appearances may occur later. Or they may not be seen until the patient comes to autopsy.

From various post-mortem reports and estimates based on clinical findings it can be stated that about one-third of all women who have carcinoma of the breast develop bone metastases. It has also been found that the more virulent the growth the greater the likelihood that metastases will occur. This explains why younger women are more prone to metastatic involvement. It is, therefore, of prime importance that follow-up should be frequent, and that patients should be warned to report if persistent pain is experienced.

Pain without demonstrable X-ray appearances should be regarded as clinical evidence of metastasis if persisting after a month.

*X-ray therapy* is given for:

1. Relief of pain, bringing with it a feeling of improvement in general health and an increase of the patient's confidence.

2. Prolongation of life.

3. Healing of fractures (this occurs frequently).

4. Relief of paraplegia. This occurs only rarely and usually in early cases.

The most valuable type of radiation therapy is that known as Deep X-ray (usually 220 to 250 kv.). Cases may be divided into those with single or those with multiple metastases.

For single metastases we give either a course lasting two to three weeks to the affected site, or a large single application in cases where the pain is excruciating. For multiple areas wide treatment may be given, e.g. to the whole of the pelvis or treatment given to the painful areas. Adequate treatment will be tolerated because of the patient's good general condition. The degree of radiation sickness is negligible.

*Results.* X-ray therapy will succeed where opiates are only temporarily successful. The patient and her relatives feel that something useful (and it is useful) is being done. While metastases are confined to the bones the prognosis remains good. Involvement of the lungs and liver usually makes the outlook hopeless. The benefits of treatment may be experienced within two to three days, or may take much longer to become established. X-ray pictures may show evidence of regression of growth and healing of bone.

X-ray therapy may act as follows:

1. Complete relief.

2. Considerable relief allowing a bedridden patient to get up; a residual ache may remain.

3. Partial relief, i.e. less pain but not sufficient amelioration of symptoms to allow patient to get up.

4. No relief. The failures are usually associated with inadequate treatment.

The number of cases which have been treated at Bristol do not yet lend themselves to statistical analysis. In any case statistics do not always tell the whole story. I am going to quote one case only.

A married woman aged 48 operated upon in 1946 for an advanced carcinoma of left breast with involvement of skin and axillary glands: treated with deep X-rays. Very well until March, 1948, when she developed symptoms of metastases in the pelvic bones. A month later these were demonstrable by X-rays and she was given a course of deep X-ray therapy. Yet a month later her pain had gone, and she was walking better: and in November (i.e. six months later) there was marked sclerosis in the treated area. Recent X-rays now show density in the place of previous osteolytic deposits. She has no symptoms, feels and looks perfectly fit. This patient is a keen gardener, and the fact that prior to her X-ray therapy to the metastases she was unable to do any gardening only added to her misery.

It would obviously be wrong to make claims in one case alone: but this one is not atypical, neither in my experience nor in that of many other radiotherapists. X-ray therapy is not the last word on this subject, but in the light of our present knowledge it is the best we have to offer. It can restore a dispirited and desperately ill woman to full health, giving her hope and courage to carry on her normal life. Even in those patients doomed to die of the disease, it is better to give a few months (or years) of ease and comfort, than to resort immediately to morphinization with all its attendant horrors.

### REFERENCES

1. Osler, W. (1906), "Medical Aspects of Cancer of Breast", *B.M.J.* **1**. 1.

2. Ewing, James, *Neoplastic Diseases*, Fourth Ed., 1941.

3. Burch, H. A. (1944), "Osseous Metastases from Graded Cancers of the Breast", *Am. J. Roentgenol & Radiotherapy*, **52**. 1.

4. Bouchard, J. (1945), "Skeletal Metastases in Cancer of the Breast", *Am. J. Roentgenology & Radiotherapy*, **54**. 156-171.

